# Comprehensively analyzing the genetic alterations, and identifying key genes in ovarian cancer

**DOI:** 10.32604/or.2023.028548

**Published:** 2023-04-10

**Authors:** QINGLING TANG, WARDA ATIQ, SHAISTA MAHNOOR, MOSTAFA A. ABDEL-MAKSOUD, MOHAMMED AUFY, HAMID YAZ, JIANYU ZHU

**Affiliations:** 1 Department of Gynecology and Obstetrics, Shanghai Songjiang District Jiuting Hospital, Shanghai, 20000, China; 2 Department of Medicine, Fatima Jinnah Medical University, Lahore, 42000, Pakistan; 3 Department of Botany and Microbiology, College of Science, King Saud University, Riyadh, 11451, Saudi Arabia; 4 Department of Pharmaceutical Sciences, Division of Pharmacology and Toxicology, University of Vienna, Vienna, 1010, Austria; 5 Department of Trauma Surgery, The First Affiliated Hospital of Wenzhou Medical University, Wenzhou, 325000, China

**Keywords:** Ovarian cancer, Prognosis, Diagnosis, Therapeutic targets

## Abstract

Though significant improvements have been made in the treatment methods for ovarian cancer (OC), the prognosis for OC patients is still poor. Exploring hub genes associated with the development of OC and utilizing them as appropriate potential biomarkers or therapeutic targets is highly valuable. In this study, the differentially expressed genes (DEGs) were identified from an independent GSE69428 Gene Expression Omnibus (GEO) dataset between OC and control samples. The DEGs were processed to construct the protein-protein interaction (PPI) network using STRING. Later, hub genes were identified through Cytohubba analysis of the Cytoscape. Expression and survival profiling of the hub genes were validated using GEPIA, OncoDB, and GENT2. For exploring promoter methylation levels and genetic alterations in hub genes, MEXPRESS and cBioPortal were utilized, respectively. Moreover, DAVID, HPA, TIMER, CancerSEA, ENCORI, DrugBank, and GSCAlite were used for gene enrichment analysis, subcellular localization analysis, immune cell infiltration analysis, exploring correlations between hub genes and different diverse states, lncRNA-miRNA-mRNA co-regulatory network analysis, predicting hub gene-associated drugs, and conducting drug sensitivity analysis, respectively. In total, 8947 DEGs were found between OC and normal samples in GSE69428. After STRING and Cytohubba analysis, 4 hub genes including TTK (TTK Protein Kinase), (BUB1 mitotic checkpoint serine/threonine kinase B) BUB1B, (Nucleolar and spindle-associated protein 1) NUSAP1, and (ZW10 interacting kinetochore protein) ZWINT were selected as the hub genes. Further, it was validated that these 4 hub genes were significantly up-regulated in OC samples compared to normal controls, but overexpression of these genes was not associated with overall survival (OS). However, genetic alterations in those genes were found to be linked with OS and disease-free (DFS) survival. Moreover, this study also revealed some novel links between TTK, BUB1B, NUSAP1, and ZWINT overexpression and promoter methylation status, immune cell infiltration, miRNAs, gene enrichment terms, and various chemotherapeutic drugs. Four hub genes, including TTK, BUB1B, NUSAP1, and ZWINT, were revealed as tumor-promotive factors in OC, having the potential to be utilized as novel biomarkers and therapeutic targets for OC management.

## Introduction

Ovarian cancer (OC) is the leading cause of mortality among all gynecologic cancers [1,2]. Additionally, the majority of OC patients are diagnosed in the late stages [3,4]. Even after successful cytoreductive surgery (CRS) in combination with platinum-based chemotherapy, most OC patients still relapse [5]. The 5-year survival rate for OC patients suffering from advanced stages is nearly 30% [6]. Therefore, exploring promising novel molecular biomarkers and gaining an understanding of the different oncogenes involving in the pathogenesis of OC is of great importance.

Gene expression profiling is an effective technique for identifying differentially expressed genes (DEGs) among patients and healthy control groups [7]. DEGs are useful for investigating molecular signaling pathways and examining regulatory networks connected to those pathways across various cancers, including OC. Currently, numerous DEGs have been reported in the medical literature that can be linked to the development and progression of OC [8–10]. However, the results of those reports are inconsistent because of tissue heterogeneity, the utilization of different sample sizes, and bioinformatics analysis for the detection of DEGs. Moreover, the outcome of an individual experiment for exploring DEGs between OC and normal sample groups has a high risk of bias; therefore, exploring DEGs using integrated analyses based on multiple expression databases could help to enhance the representativeness and reliability of the results.

The microarray technique is a widely used technique for documenting variations in the expression of various vital genes, such as oncogenes and tumor suppressor genes [11]. Using microarray technology, researchers around the world have published a substantial amount of data on various publicly available platforms, and among those, the Gene Expression Omnibus (GEO) database is the most important [12]. Numerous studies have been published in the medical literature that used bioinformatics analyses for identifying DEGs in OC based on microarray GEO datasets. For instance, Yang et al. explored the 17 most important OC-related DEGs with the help of the protein-protein interaction (PPI) network [13]. Besides this, Lou et al. discovered the three most important DEGs, including GJB2, S100A2, and SPOCK2, which were significantly overexpressed in OC patients with an advanced stage compared with early-stage OC patients [14]. In this study, they also highlighted a co-regulatory role of the lncRNA-hsa-miR-363-3p-SPOCK2 pathway in the development of OC [14].

In the current research, GSE69428 [15] microarray-based dataset was retrieved from the GEO database and analyzed for obtaining DEGs between OC and the normal sample group. Later, with the help of the Cytohubba plug-in, a total of four genes were shortlisted as hub genes. Then, the potential roles of the identified hub genes in OC development and progression were explored and validated, and their associations with OC patient survival duration were identified using a detailed multi-layered methodology. Finally, functional enrichment analyses were implemented to uncover the underpinning molecular mechanisms behind the pathogenesis of OC. We believe that the outcomes of the present study could be valuable for giving an insight into the more valuable targets for exploring molecular mechanisms and providing effective treatment methods in OC.

## Materials and Methods

### Data collection and preprocessing

Using the keywords “Ovarian cancer,” and “Ovarian neoplasm”, the GEO database (https://www.ncbi.nlm.nih.gov/geo/) was searched comprehensively. After a brief search, the GSE69428 [15] dataset was selected as the experimental dataset, which includes 10 normal samples and 19 OC patient samples. Moreover, before analysis, we screened gene probes in the selected dataset. All gene probes which does not have corresponding genes in the dataset, the expression data of these gene probes were removed. In addition to this, if a gene has two or more probes, the average expression of all these probes was retained.

### Identification of DEGs

The DEGs among the OC and control sample groups were identified with the help of the R language “Limma” package. The following selection criteria were adopted to screen the DEGs: |log2FC| > 1.3, and *p* (*t*-test, student) < 0.05 [16,17]. The Fold Change (FC) in expression highlights the considerable differences among DEGs.

### Construction of PPI, module identification, and the selection of hub genes

In total, 250 genes with the highest expression variations in terms of *p*-values were shortlisted for further analysis. The PPI of the shortlisted genes was constructed with the help of the STRNG database [18]. The constructed PPI was subjected to MCODE analysis [19] using the Cytoscape tool [20] for the identification of key module. The key module was screened through the Cytohubba function [21] in the Cytoscape tool for selecting hub genes. Based on the 4 different scoring algorithms, the maximum neighborhood component (MNC), the density of the maximum neighborhood component (DMNC), the maximal clique centrality (MCC), and the degree of the Cytohubba [22], the shared top four genes by these algorithms were selected as hub genes.

### GEPIA database

The GEPIA database is the latest online web-based tool that enables users to perform interactive and customizable analyses between normal-v-normal cancer samples, including differential gene expression profiling, correlation analysis, survival analysis, and plotting gene expression plots based on different pathological stages using Cancer Genome Atlas (TCGA) expression data [23]. This database was used in our study with default settings to verify the expression of hub genes, and perform survival, and correlation analysis in OC and normal samples.

### OncoDB and GENT2 databases

Then, to further validate hub genes’ expression across OC tissues and cell lines, we employed the OncoDb (https://oncodb.org/genomic_profile_methyl_non_virus.html) [24] and GENT2 (http://gent2.appex.kr/gent2/) [25] databases. Both of these databases contain expression data of normal and cancer tissue samples from microarray experiments. For OncoDB and GENT2 all hub gene queries were performed with default settings, and expression results were presented in the form of box plots.

#### MEXPRESS

The MEXPRESS database (https://mexpress.be) in-house clinical, and DNA methylation data for all human genes from The Cancer Genome Atlas (TCGA) project [26]. The MEXPRESS was conducted with default setting in our study to check the DNA promoter methylation level of identified hub genes in OC.

### cBioPortal

The cBioPortal (https://www.cbioportal.org/), an online open-access resource, is used for conducting multidimensional cancer genomic analyses on TCGA cancer datasets [27]. In this study, a TCGA OC dataset, namely, “TCGA Nature 2011 (563 cases),” was used for analyzing genetic mutations, mutational hotspots, co-expressed genes, and the effect of mutations on the survival and mRNA expression levels of the hub genes in OC.

### The human protein atlas (HPA)

The HPA online database (https://www.proteinatlas.org/) [28] was used in the present study to find the subcellular localization of proteins encoded by the hub genes in OV cells. Moreover, this database has also helped to perform hub genes expression and survival analysis at protein level.

### Functional enrichment analysis

The GSEA tool was used in this study to perform functional enrichment, including GO and KEGG analysis of the hub genes, with a *p*-value of 0.05. This tool associate GO and KEGG terms to a group of specified genes/proteins and identified KEGG and GO terms based on the biological function relevancy [29].

### TIMER database

The TIMER database (http://timer.cistrome.org/) has a web-based interface and is used to evaluate the tumor infiltration of immune cells [30]. This is a comprehensive database that in-house immune infiltration data of different types of immune cells in a variety of cancers. In this study, the expression of hub genes was plotted against immune cell infiltration levels in OC.

### CancerSEA analysis

CancerSEA (http://biocc.hrbmu.edu.cn/CancerSEA/) was developed for decoding Pearson correlations between gene(s) of interest and 14 different functional states at the single-cell level in human cancers [31]. Herein, we utilized CancerSEA to explore the correlations between hub genes and different functional states in OC.

### miRNA network of the hub genes

ENCORI (https://starbase.sysu.edu.cn/) is a publicly available resource for analyzing interactions between mRNAs, miRNAs, and lncRNAs among 23 species [32]. In our study, the miRNA network of the identified hub genes was predicted using the ENCORI database with default settings.

### Hub genes’ drug prediction analysis

The identified hub genes can be promising therapeutic targets, and in this view, we conducted the DrugBank (https://go.drugbank.com/) analysis to identify hub genes’ associated drugs. This database provides details from different reliable sources on drugs targeting hub genes [33].

GSCALite (http://bioinfo.life.hust.edu.cn/web/GSCALite/) is an online analysis tool for conducting gene-set cancer analysis [33]. In this study, we applied the GSCALite tool for analyzing hub genes drug sensitivity, which may help to select more appropriate drugs against the hub genes for targeted therapy.

### Statistics analysis

DEGs were identified using a *t*-test [34]. While for GO and KEGG enrichment analysis, we used Fisher’s Exact test for computing statistical difference [35]. Correlational analyses were carried out using Pearson method. For comparison, a student *t*-test was adopted in the current study. All the analyses were carried out in R version 3.6.3.

## Results

### Screening of DEGs

**Figure 1:**
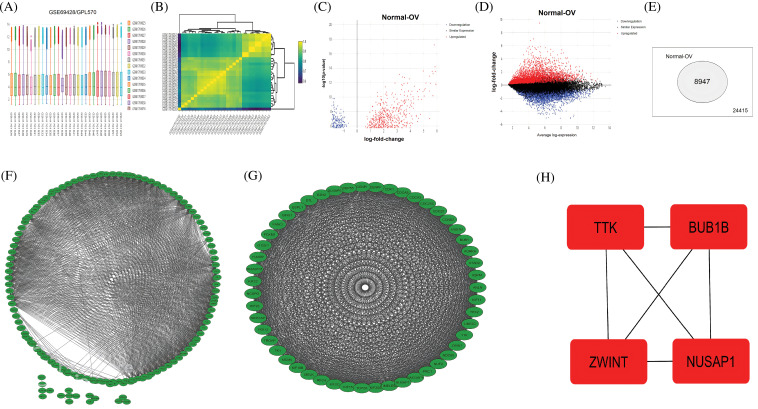
A comparison between expression profiles of samples, volcano graphs of DEGs, a total count of DEGs, PPI networks, and the identification of hub genes from the GSE69428 microarray dataset. (A and B) A comparison between expression profiles of samples in the GSE69428 microarray dataset, (C and D) Volcano graphs of the DEGs observed in the GSE69428 microarray dataset, (E) A total count of DEGs and non-DEGs in the GSE69428 microarray dataset, (F) A PPI network of the top 250 DEGs in the GSE69428 microarray dataset, (G) A PPI network of the most significant module, and (H) Identified four hub genes.

Using the R package “limma” with |log2FC| > 1.3, and *p*-value < 0.05 cutoff criterion, the DEGs were screened among OC (n = 19) and normal samples (n = 10) included in the GSE69428 dataset (Figs. 1A and 1B). As a result, a total of 8947 DEGs were identified (Figs. 1C–1E). Based on the *p*-value, the top 250 DEGs in terms of the level of significance were selected for further analysis in the present study.

### PPI network construction, module identification, and hub genes exploration

The identified 250 DEGs were subjected to STRING analysis to construct the PPI, using a minimum required interaction score > 0.4 as a threshold (Fig. 1F). The constructed PPI network had 250 nodes and 454 edges (Fig. 1F). Next, to screen the top genes associated with OC development, we identify the most significant modules within the constructed PPI. As shown in Fig. 1G, the identified module was the most significant module in terms of total gene count (n = 32). Therefore, we further process this module for hub genes exploration.

To explore a few important hub genes in OC patients, we combined 4 different scoring algorithms, including MNC, DMNC, MCC, and the degree of the Cytohubba [22]. The top four DEGs shared by these algorithms were regarded as hub genes. The results showed that based on these algorithms, the 4 significantly up-regulated hub genes include TTK (TTK Protein Kinase), (BUB1 mitotic checkpoint serine/threonine kinase B) BUB1B, (Nucleolar and spindle-associated protein 1) NUSAP1, and (ZW10 interacting kinetochore protein) ZWINT (Fig. 1H).

### Hub genes expression profiling, correlation analysis, and prognostic values in GEPIA

**Figure 2:**
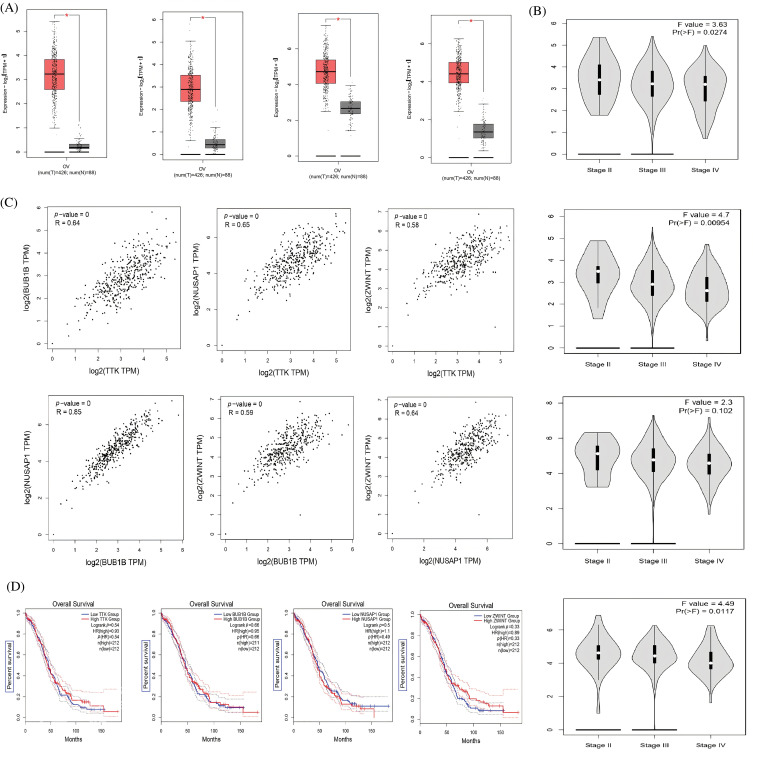
Expression profiling, Pearson correlation, and survival analysis of TTK, BUB1B, NUSAP1, and ZWINT via GEPIA. (A) Expression profiling of TTK, BUB1B, NUSAP1, and ZWINT in OC and normal controls, (B) Expression profiling of TTK, BUB1B, NUSAP1, and ZWINT in OC samples of different cancer stages, (C) Pearson correlation analysis of TTK, BUB1B, NUSAP1, and ZWINT in OC samples, and (D) OS survival analysis of TTK, BUB1B, NUSAP1, and ZWINT in OC patients.

Since 4 up-regulated genes were selected as hub genes (TTK, BUB1B, NUSAP1, and ZWINT) out of the 250 DEGs, we then first performed the expression analysis of these genes using the GEPIA database. Results showed that hub gene expressions were up-regulated in OC samples relative to controls (Fig. 2A). These results further highlighted that hub gene expression varies greatly among OC patients of different cancer stages (Stage II, Stage III, and Stage IV) (Fig. 2A). Our results from the GEPIA database are in line with the expression results of the analyzed dataset (GSE69428). Then, since hub genes (TTK, BUB1B, NUSAP1, and ZWINT) are significantly overexpressed, we subjected these genes to Pearson correlation analysis using the “Correlation” feature of the GEPIA database. The results of the correlation analysis highlighted that these genes had positive correlations with each other (Fig. 2C). Next, we explored the prognostic values of the hub genes in OC via the survival analysis feature of the GEPIA. The results showed the identified hub genes had insignificant prognostic values in OC samples having higher expressions of these genes (Fig. 2D). Thus, these hub genes could not accurately predict the survival rates of OC patients.

### Verification of the hub genes expression pattern via the OncoDB and GENT2

**Figure 3:**
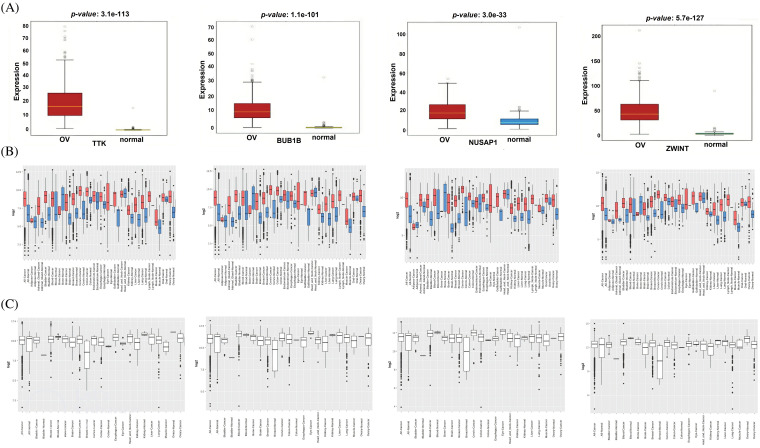
Expression validation of TTK, BUB1B, NUSAP1, and ZWINT via the OncoDB and GENT2 databases. (A) Expression validation of TTK, BUB1B, NUSAP1, and ZWINT in OC and normal control samples via the OncoDB database, (B) Expression validation of TTK, BUB1B, NUSAP1, and ZWINT in OC and normal control samples via the GENT2 database, and (C) Expression validation of TTK, BUB1B, NUSAP1, and ZWINT in OC and normal control cell lines via the GENT2 database.

Additionally, we performed the expression validation analysis of the hub genes in TCGA datasets using the OncoDB and GENT databases. As shown in Figs. 3A and 3B, the mRNA expressions of TTK, BUB1B, NUSAP1, and ZWINT were significantly higher in OC samples relative to the normal individuals. Moreover, mRNA expressions of the hub genes were also found to be significantly up-regulated in OC cell lines as compared to normal individual cell lines via the GENT2 database (Fig. 3C). Therefore, it is obvious to say that TTK, BUB1B, NUSAP1, and ZWINT hub genes are significantly overexpressed in OC patient clinical samples as well as cell line samples.

### Promoter methylation is not correlated with the expression profiles of TTK, BUB1B, NUSAP1, and ZWINT

**Figure 4:**
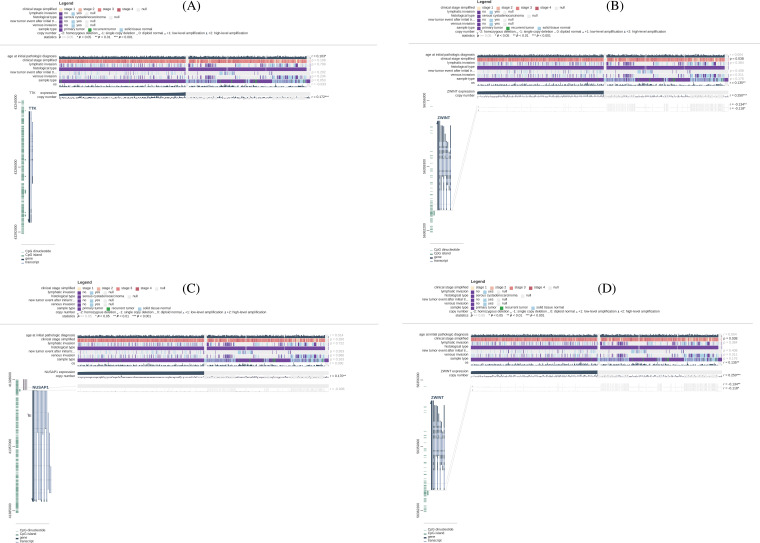
Methylation status exploration of TTK, BUB1B, NUSAP1, and ZWINT via the MEXPRESS in OC and normal samples. (A) TTK, (B) BUB1B, (C) NUSAP1, and (D) ZWINT.

We assessed the involvement of promoter methylation in the dysregulation of TTK, BUB1B, NUSAP1, and ZWINT hub genes’ expression in OC. With the help of MEXPRESS, it was explored if the TTK, BUB1B, NUSAP1, and ZWINT expressions at the mRNA level were regulated by the promoter methylation in OC or not. Interestingly, owing to the promoter methylation level, we found the significant hypermethylation of TTK, BUB1B, NUSAP1, and ZWINT genes’ promoter in OC samples than in controls (Fig. 4). Therefore, it is concluded that the higher expressions of TTK, BUB1B, NUSAP1, and ZWINT were not related to the promoter methylation levels of these genes.

### Genetic mutations, mutational hotspots, co-expressed genes, and the effect of mutations on the survival and mRNA expression levels of the hub genes

**Figure 5:**
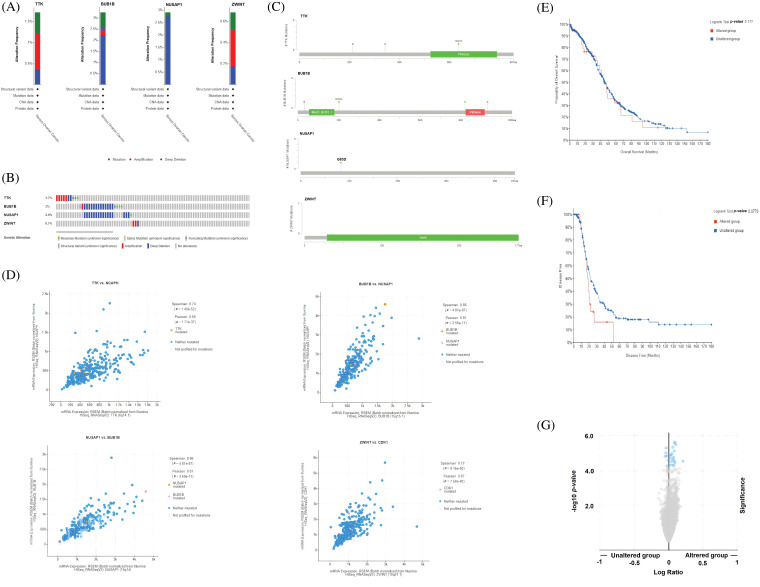
Exploration of genetic alteration frequencies, mutational hotspots, co-expressed genes, OS, DFS analyses, and the effect of genetic mutations on the expression of TTK, BUB1B, NUSAP1, and ZWINT in OC via the cBioPortal. (A and B) Types and frequencies of the genetic alterations in TTK, BUB1B, NUSAP1, and ZWINT, (C) Location of the observed mutations in TTK, BUB1B, NUSAP1, and ZWINT in the encoded proteins, (D) Identification of co-expressed genes with TTK, BUB1B, NUSAP1, and ZWINT, (E and F) OS and DFS analysis of TTK, BUB1B, NUSAP1, and ZWINT in genetically altered and unaltered OC group, and (G) Effect of the genetic alterations in TTK, BUB1B, NUSAP1, and ZWINT on the gene expression in genetically altered and unaltered OC group.

Genetic mutations, mutational hotspots, co-expressed genes, and the effect of mutations on the survival and mRNA expression levels of the TTK, BUB1B, NUSAP1, and ZWINT were explored in OC patients using the cBioPortal database. BUB1B gene was the most genetically altered gene and was altered in 3% samples of 513 analyzed total samples (Figs. 5A and 5B). The alteration rates of NUSAP1, TTK, and BUB1B were 2.9%, 1.7%, and 0.7%, respectively, in the analyzed OC samples, and the deep deletion accounted for most of the changes in those genes (Figs. 5A and 5B). Concerning mutational hotspots in proteins encoded by the TTK, BUB1B, NUSAP1, and ZWINT hub genes, the D637R mutation was found to hit the functionally important domain (Pkinase) of the TTK protein (Fig. 5C), while no other mutation was found hitting functionally important domains in the BUB1B (mad3_BUB1 and Pkinase domains), NUSAP1, and ZWINT (Zwint domain) proteins (Fig. 5C). Moreover, by performing co-expressed gene analysis, we calculated correlation coefficients and identified that along with TKK, NCAPH is a significant co-expressed gene in OC samples (Fig. 5C), while BUB1B and NUSAP1 are also highly co-expressed genes in OC samples, and for ZWINT, the highly co-expressed gene was CDK1 (Fig. 5C). Using the “Survival analysis” feature of the cBioPortal database, we drew the OS and DFS curves of the hub genes between the two sample groups, i.e., one group consisting of those OC samples which were genetically altered with hub gene alterations and the second group consisting of those samples that did not have alterations in the hub genes (Figs. 5E and 5F). Results of the survival analysis revealed that the genetically altered group of OC samples had the worst OS and DFS survival rates relative to the unaltered group of OC patients (Figs. 5E and 5F). However, the results were insignificant. Next, we further evaluated whether OC samples with mutated hub genes have overall higher expression or not via the cBioPortal database. As a result, it was noted that hub gene-mutated OC samples had higher overall expression relative to the non-mutated group of OC samples (Fig. 5G).

### Exploring subcellular localizations of TTK, BUB1B, NUSAP1, and ZWINT through HPA database

**Figure 6:**
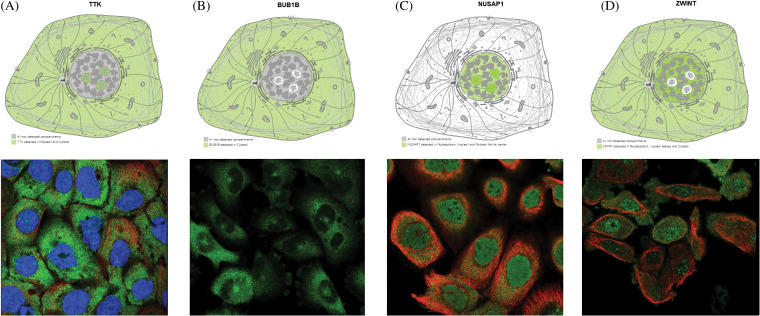
Predicting subcellular localization of TTK, BUB1B, NUSAP1, and ZWINT in OC tissue via the HPA database. (A) TTK, (B) BUB1B, (C) NUSAP1, and (D) ZWINT.

Through the HPA database, the subcellular locations of TTK, BUB1B, NUSAP1, and ZWINT were explored across OC cells. For TTK, this protein was mainly enriched in the neucleoli and cytosol (Fig. 6A). The BUB1B localization was found in the cytosol (Fig. 6B), while the NUSAP1 localization was enriched in the neucleoli and neucleoli fibrillar center (Fig. 6C), and the ZWINT localization was seen in the nucleoplasm, nuclear bodies, and cytosol (Fig. 6D).

### Functional enrichment analysis

**Figure 7:**
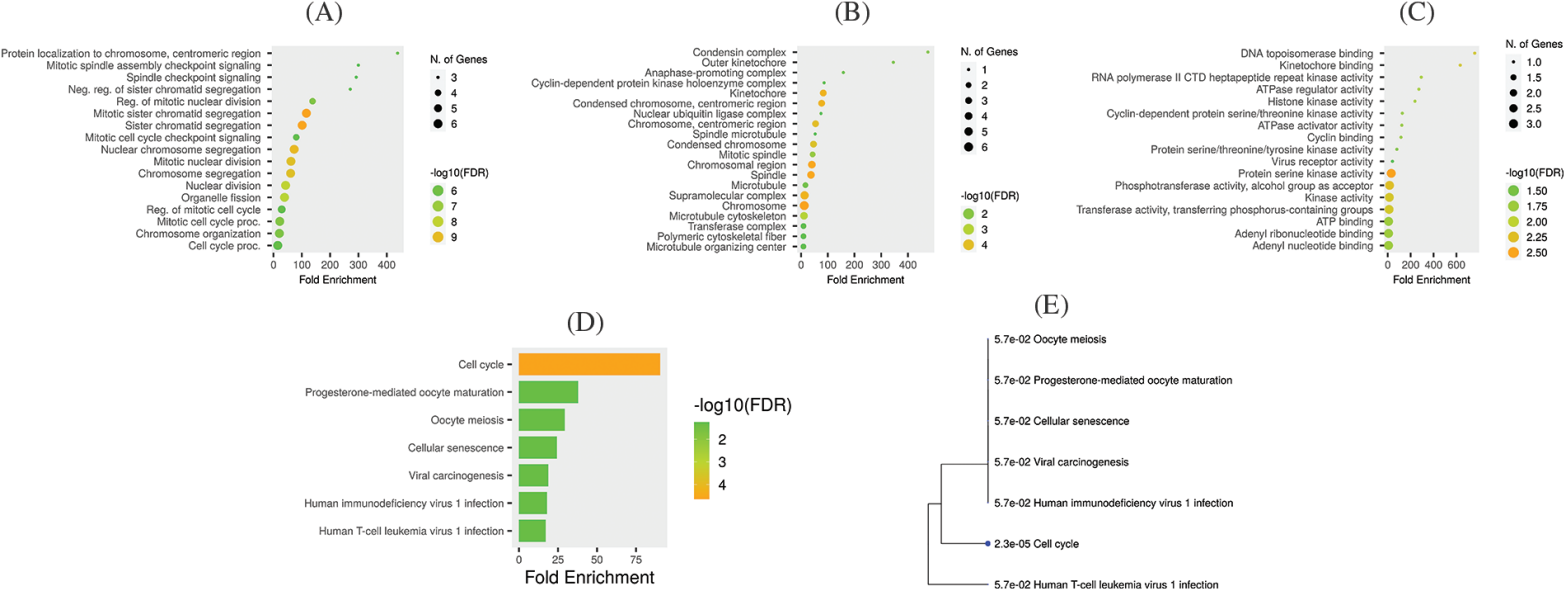
Gene enrichment analysis of TTK, BUB1B, NUSAP1, and ZWINT. (A) TTK, BUB1B, NUSAP1, and ZWINT associated CC terms, (B) TTK, BUB1B, NUSAP1, and ZWINT associated MF terms, (C) TTK, BUB1B, NUSAP1, and ZWINT associated BP terms, (D) TTK, BUB1B, NUSAP1, and ZWINT associated KEGG terms, and (E) KEGG terms phylogram.

GO and KEGG enrichment analyses of the hub genes (TTK, BUB1B, NUSAP1, and ZWINT) were done with the help of the GSEA tool. Cellular components (CC), biological process (BP), and molecular functions (MF) are 3 major functions of the GO enrichment analysis. In this study, protein localization to chromosome centromeric region, mitotic spindle assembly checkpoint signaling, spindle check point signaling, neg. reg. of sister chromatid segregation, and reg. of mitotic nuclear division, etc., were the major CC of the hub genes (Fig. 7A). Condensing complex, outer kinetochore, anaphase promoting complex, cyclic-dependent protein kinase holoenzyme complex, and kinetochore, etc., BP were mainly associated with hub genes (Fig. 7B), while DNA topoisomerase binding, kinetochore binding, DNA polymerase II CTD heptapeptide repeat kinase activity, ATPase regular activity, and histone kinase activity, etc., were the primary MFs of the hub genes (Fig. 7C). Moreover, KEGG pathways for the identified hub genes are highlighted in Figs. 7D and 7E, and cell cycle, progesterone-mediated oocyte maturation, oocyte meiosis, cellular sentences, and viral carcinogenesis pathways were found to be involved in the pathogenesis of OC.

### Immune cell analysis of the hub genes

**Figure 8:**
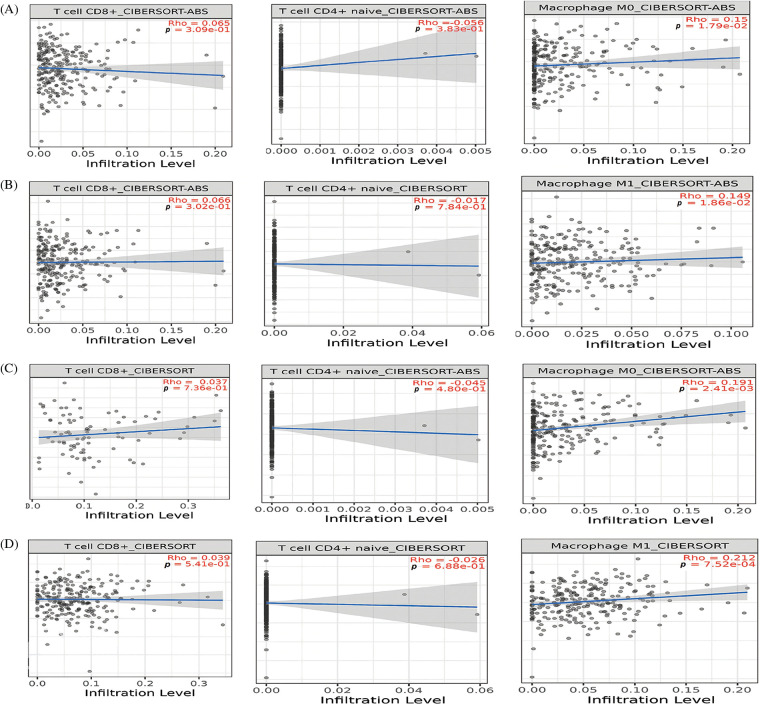
Correlation analysis of TTK, BUB1B, NUSAP1, and ZWINT hub gene expressions with different immune cell (CD8 + T, CD4 + T, and macrophages) infiltration levels. (A) TTK, (B) BUB1B, (C) NUSAP1, and (D) ZWINT.

Next, we further evaluated relationships among different immune cell infiltration (CD8 + T, CD4 + T, and macrophages) and hub gene expressions (TTK, BUB1B, NUSAP1, and ZWINT) via the “TIMER” tool. The TTK, BUB1B, NUSAP1, and ZWINT expressions were found to be positively correlated (*p* < 0.05) with the infiltration of CD8 + T and macrophages cells, while negatively correlated (*p* < 0.05) with the infiltration of CD4 + T in OC (Fig. 8).

### Single-cell functional analysis

**Figure 9:**
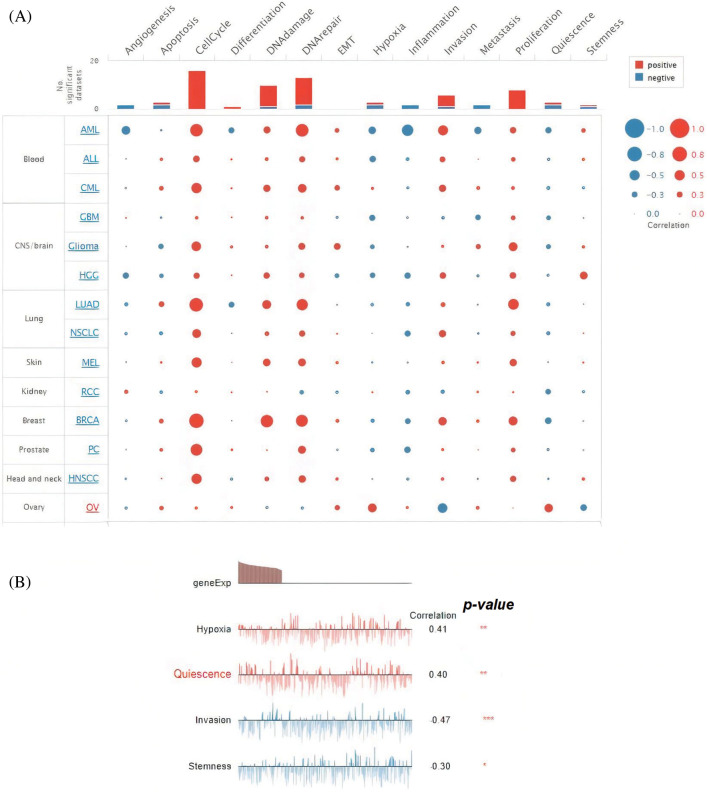
Association of TTK, BUB1B, NUSAP1, and ZWINT hub gene expressions with fourteen different states in OC. (A) Overall association (significant/insignificant) of TTK, BUB1B, NUSAP1, and ZWINT with Angiogenesis, apoptosis, cell cycle, and many others, and (B) Significant association of TTK, BUB1B, NUSAP1, and ZWINT with hypoxia, Quiescence, and others.

Hub genes’ further involvement in OC at the single cell level was explored via the CancerSEA database. All hub genes, including TTK, BUB1B, NUSAP1, and ZWINT, were revealed to be linked (positively or negatively) with the fourteen different states at the single cell level in OC (Fig. 9A). However, hub gene expressions were notably positively correlated with hypoxia and quiescence, while negatively correlated with invasion and stemness (Fig. 9B).

### lncRNA-miRNA-mRNA interaction network

**Figure 10:**
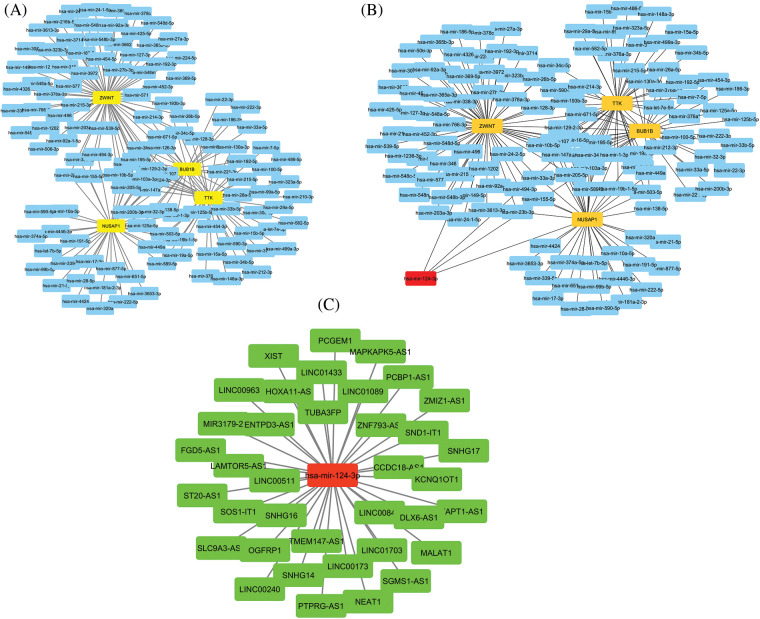
lncRNA-miRNA-mRNA co-regulatory network of the TTK, BUB1B, NUSAP1, and ZWINT hub genes. (A) A PPI of miRNAs targeting hub genes, (B) A PPI highlighting most important miRNA (has-mir-124-3p) targeting all hub genes, and (C) A PPI of lncRNAs targeting has-mir-124-3p. Yellow color nodes: mRNAs, Blue color nodes: miRNAs, Red color nodes: Most important miRNA in the network, and Green color nodes = lncRNAs.

via the ENCORI and Cytoscape, we constructed the lncRNA-miRNA-mRNA co-regulatory networks of the TTK, BUB1B, NUSAP1, and ZWINT. In the constructed networks, the total counts of lncRNAs, miRNAs, and mRNAs were 37, 152, and 4, respectively (Fig. 10). Based on the constructed networks, we identified one miRNA (has-mir-124-3p), that targets all hub genes simultaneously. Therefore, we speculate that the identified lncRNAs, hsa-mir-124-3p, and hub genes (TTK, BUB1B, NUSAP1, and ZWINT) (Fig. 10) as an axis, might also be the potential inducers of the OC.

### Drug prediction and sensitivity analysis of hub genes

**Figure 11:**
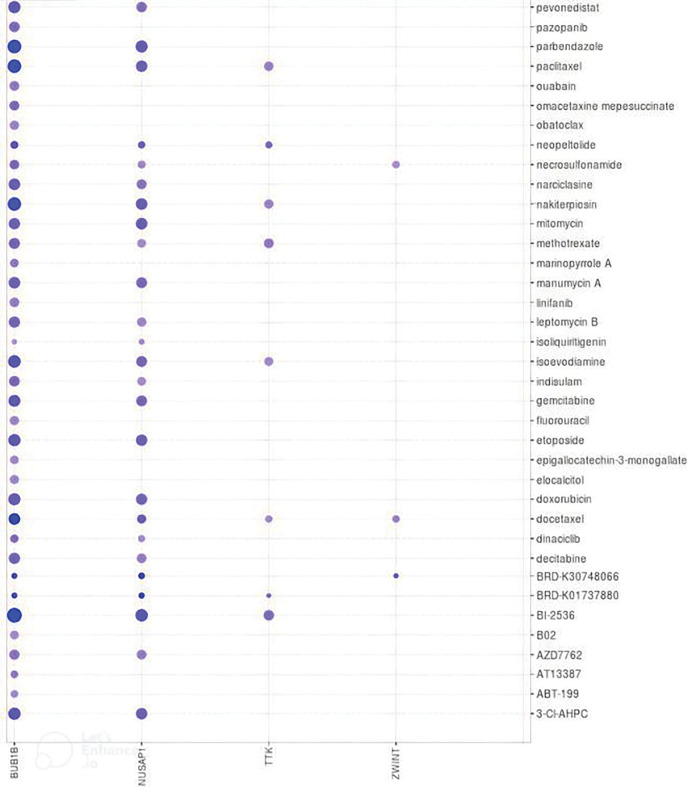
Correlation analysis of TTK, BUB1B, NUSAP1, and ZWINT expressions with the sensitivity of different chemotherapeutic drugs. Blue color represents the negative correlation.

Medical treatment is the preliminary choice to handle the disease for patients who are suffering from OC. Therefore, a selection of suitable candidate potential drugs is necessary. In the current study, with respect to the identified hub genes, we explored some suitable therapeutic drugs for the treatment of OC via the DrugBank database and also checked the drug sensitivity of various available chemotherapeutic drugs against the overexpression of TTK, BUB1B, NUSAP1, and ZWINT. As a result, it was noted that cyclosporine and bicalutamide drugs along with many other drugs, are the negative expression regulators of TTK, BUB1B, NUSAP1, and ZWINT mRNA expression (Table 1). While higher expression of these genes is positively correlated with the sensitivity of various other drugs, such as pevonidistal, pazopanib, parbendazole, etc., as highlighted in Fig. 11.

**Table 1:** DrugBank-based hub genes-associated drugs

Sr. No.	Hub gene	Drug name	Effect	Reference	Group
1	TTK	Cyclosporine	Decrease expression of TTK mRNA	A20661	Approved
		Dasatinib		A21899	
		Calcitriol		A22301	
		Etoposide		A22582	
		Bicalutamide		A21424	
2	CCNB1	Belinostat	Decrease expression of CCNB1 mRNA	A21036	Approved
		Estradiol		A21133	
		Bicalutamide		A21424	
		Cyclosporine		A20661	
3	NUSAP1	Estradiol	Decrease expression of NUSAP1 mRNA	A21098	Approved
		Bicalutamide		A21424	
		Cyclosporine		A20661	
4	ZWINT	Cyclosporine	Decrease expression of ZWINT mRNA	A20661	Approved

## Discussion

Despite substantial advancements in the currently existing methods for treating OC, such as chemotherapy, radiotherapy, surgery, and targeting novel agents, this disease is still untreatable since from past several decades. Therefore, the discovery of novel OC biomarkers and exploring their molecular mechanisms are crucial for OC prevention and treatment. Currently, bioinformatics-based analyses are playing important roles in understanding cancer biology and, in this way, facilitating the identification of novel biomarkers by integrating multi-omics data from different online accessible databases, such as the GEO database.

In the present study, we initially screened out OC-related DEGs from GSE69428 GEO datasets. A PPI network of the OC-related DEGs was constructed with help of the STRING database, and four significantly up-regulated genes (TTK, BUB1B, NUSAP1, and ZWINT) were identified as hub genes using the Cytohubba application of the Cytoscape. Then, the mRNA expression analysis, correlation analysis, and survival analysis of the hub genes were carried out in the GEPIA database. Results of the analysis indicated that all hub genes, including TTK, BUB1B, NUSAP1, and ZWINT, were up-regulated and positively correlated to each other across OC tissue samples relative to normal controls with statistical significance. Cancer stage-wise expression analysis of the hub genes revealed that the expressions of TTK, BUB1B, and NUSAP1 increased while the expression of ZWINT decreased slightly with the stage-wise continued progression of OC. The obtained survival curves further illustrated that OC patients having the higher expression of the hub genes had better survival. Therefore, we speculate that identified hub genes may not be used as potential prognostic indicators for OC patients. Moreover, the expression validation analysis of TTK, BUB1B, NUSAP1, and ZWINT via OncoDB and GENT2 in OC tissue as well as cell lines samples also confirmed the higher expression of these genes with statistical significance relative to the control samples.

The TTK gene is found on chromosome 6q13-q21 and codes for TTK protein [36,37]. This protein is very viral for the regulation of mitotic checkpoints and the attachment of chromosome [36]. It was reported earlier that the overexpression of TTK results in the enlargement of the centrosome and chromosomal instability, ultimately leading to the development of different cancers [38,39]. TTK’s potential diagnostic and prognostic importance has been reported in thyroid cancer, triple-negative breast cancer, and different types of lung cancer [40–42]. In addition to this, it was also reported that different TTK inhibitors, such as AZ3146 and MPI-0479605, can effectively be utilized to inhibit the proliferation potential of hCt-116 colon cancer cells, showing the overexpression of TTK [43–45].

The BUB1B belongs to the spindle assembly checkpoint (SAC) protein family [46,47], which prevents the separation of premature sister chromatids during mitosis until all kinetochores get attached properly to the mitotic spindle [48]. Concerning BUB1B’s role in the SAC family, this protein is very important in SAC signaling for stabilizing kinetochores’ attachment to spindle microtubules [49,50]. *Huang* et al. [51] have shown in their study that BUB1B binds directly with Cdc20, MAD2, and BUB3 proteins for constituting a mitotic checkpoint complex, required for the inhibition of anaphase-promoting complex (APC/C) during mitosis [52,53]. Concerning BUB1B’s role in the APC/C complex, this protein ensures proper chromosome segregation by suppressing the onset of anaphase [54]. Given the important roles of BUB1B in mitosis, the dysregulation of BUB1B is often found to result in aneuploidy and chromosomal instabilities, ultimately increasing the chances of cancer development [55–57].

NUSAP1 is a cell cycle-regulating protein that is involved in spindle formation [58,59]. The accurate spindle formation ensures the correct chromosomal division, which is critical for the normal cell division process. Abnormalities in the spindle structure may lead to abnormal chromosome separation (chromosomal instability), which ultimately causes the development of cancer [60]. Recently, it was shown by several studies that NUSAP1 dysregulation has important oncogenic roles in various cancers, such as colorectal [61], prostate [62], breast [63], lung [64,65], and cervical cancer [66]. The higher expression of NUSAP1 is mainly associated with the poor prognosis of melanoma and breast-invasive carcinoma [62,67]. In addition to this, NUSAP1 overexpression was also found to promote the growth, migration, and invasion of the MCF-7 cells [68].

ZWINT is the part of the kinetochore complex that is required for the mitotic spindle checkpoint in mitotic maintenance [69]. The kinetochore performs various essential activities in the cell division process [70]. ZWINT protein is majorly involved in kinetochore functioning, mainly by regulating the association among ZW10 and centromere complexes during mitotic prometaphase [71]. Abnormalities in the mitosis process are known as the common hallmark of various malignancies, including cancer. Although the exact nature of interactions among different kinetochore components in cancer development is largely unknown, recent evidence suggests that ZWINT is overexpressed in different human cancers and is associated with the worst prognosis and early recurrence of cancer [72–74].

Earlier, the dysregulation of AURKA, CENPF, and TOP2A hub genes in OC was well documented by various previous reports [75–78]. The dysregulation of KIF11 and KIF23 in OC is less understood [79]. Another study reported that COL4A1, SDC1, and CDKN2A hub genes were highly expressed at both mRNA and protein levels in OC samples relative to normal controls [80]. One more study explored that COL6A3, CRISPLD2, and SERPINF1 hub genes were overexpressed in OC patients compared to normal samples [81].

Furthermore, we also explored that the overall genetic alterations in TTK, BUB1B, NUSAP1, and ZWINT hub genes were significantly associated with the higher expression of these genes and unfavorable OS and DFS in OC patients. However, our study failed to reveal the significant negative correlations among the expression and promoter methylation levels of TTK, BUB1B, NUSAP1, and ZWINT hub genes across OC patients, suggesting that promoter methylation level dysregulation might not be the expression regulatory mechanism of the TTK, BUB1B, NUSAP1, and ZWINT dysregulations in the OC.

In this study, we further explored that TTK, BUB1B, NUSAP1, and ZWINT hub genes were part of many diverse GO terms and were associated with different cancer-related signaling pathways, including “cell cycle, progesterone-mediated oocyte maturation, oocyte meiosis, cellular senesces, and viral carcinogenesis” pathways in the OC patients. The oncogenic roles of these pathways were earlier reported by various studies [82–85]. It was also noted in our research that the expressions of TTK, BUB1B, NUSAP1, and ZWINT hub genes are regulated simultaneously by hsa-mir-124-3p miRNA in the OC patients and higher expressions of these genes are significantly related to the immune cell infiltration (CD8 + T, CD4 + T, and macrophages). Earlier studies have shown that miR-124-3p is dysregulated across multiple human cancers, such as the cancers of the breast, bladder, retinoblastoma, glioblastoma, and esophageal [86–88]. In addition to this, many other studies have highlighted the tumor suppressor role of miR-124-3p in bladder cancer [89,90]. However, any tumor suppressor or tumor-causing role of miR-124-3p in OC is not reported anywhere. Therefore, to the best of our knowledge, this study is the first to report the probable cancer-driving role of the hsa-mir-124-3p miRNA with respect to TTK, BUB1B, NUSAP1, and ZWINT hub genes in OC.

Lastly, we identified and checked the sensitivity of various chemotherapeutic drugs against TTK, BUB1B, NUSAP1, and ZWINT expressions using the DrugBank and GSCALite databases, which could be used for treating OC patients. The details of the drugs are given in Table 1, and best to our knowledge, such potential drugs against TTK, BUB1B, NUSAP1, and ZWINT hub genes were found for the first time through this study.

Although the outcomes of this study have many merits, limitations are unavoidable. Our findings were mainly based on bioinformatics analyses. Validating the TTK, BUB1B, NUSAP1, and ZWINT using in-house clinical samples could great strengthen our findings.

## Conclusion

The findings of this study showed that four genes, including TTK, BUB1B, NUSAP1, and ZWINT, could be potential indicators for future OC diagnosis, prognosis, and new therapeutic targets. However, further research involving wet-lab experiments is needed to confirm our findings.

## Data Availability

The dataset analyzed in the current study can be found at https://www.ncbi.nlm.nih.gov/geo/.
